# Cathepsin G Is Expressed by Acute Lymphoblastic Leukemia and Is a Potential Immunotherapeutic Target

**DOI:** 10.3389/fimmu.2017.01975

**Published:** 2018-01-25

**Authors:** Maliha Khan, Selena Carmona, Pariya Sukhumalchandra, Jason Roszik, Anne Philips, Alexander A. Perakis, Celine Kerros, Mao Zhang, Na Qiao, Lisa S. St. John, Madhushree Zope, Jonathan Goldberg, Mariam Qazilbash, Haroon Jakher, Karen Clise-Dwyer, Yihua Qiu, Elizabeth A. Mittendorf, Jeffrey J. Molldrem, Steven M. Kornblau, Gheath Alatrash

**Affiliations:** ^1^Department of Leukemia, MD Anderson Cancer Center, Houston, TX, United States; ^2^Department of Stem Cell Transplantation and Cellular Therapy, MD Anderson Cancer Center, Houston, TX, United States; ^3^Department of Melanoma Medical Oncology, MD Anderson Cancer Center, Houston, TX, United States; ^4^Department of Genomic Medicine, MD Anderson Cancer Center, Houston, TX, United States; ^5^Surgical Oncology, MD Anderson Cancer Center, Houston, TX, United States

**Keywords:** cathespin G, immunotherapy, nonameric peptide, acute lymphoblastic leukemia, cross-presentation, antigens, targeted therapy, serine proteases

## Abstract

Cathepsin G (CG) is a myeloid azurophil granule protease that is highly expressed by acute myeloid leukemia (AML) blasts and leukemia stem cells. We previously identified CG1 (FLLPTGAEA), a human leukocyte antigen-A2-restricted nonameric peptide derived from CG, as an immunogenic target in AML. In this report, we aimed to assess the level of CG expression in acute lymphoid leukemia (ALL) and its potential as an immunotherapeutic target in ALL. Using RT-PCR and western blots, we identified CG mRNA and protein, respectively, in B-ALL patient samples and cell lines. We also examined CG expression in a large cohort of 130 patients with ALL *via* reverse-phase protein array (RPPA). Our data show that CG is widely expressed by ALL and is a poor prognosticator. In addition to endogenous expression, we also provide evidence that CG can be taken up by ALL cells. Finally, we demonstrate that patient ALL can be lysed by CG1-specific cytotoxic T lymphocytes *in vitro*. Together, these data show high expression of CG by ALL and implicate CG as a target for immunotherapy in ALL.

## Introduction

Patients with relapsed and refractory acute lymphoblastic leukemia (ALL) have a very aggressive malignancy with poor outcomes. Although the outcome of pediatric patients with ALL is excellent, the overall survival (OS) for adult patients with ALL is poor, especially in patients who relapse after initial therapy (1–3). Although hematopoietic stem cell transplantation (HSCT) provides cures in some cases, the 1-year OS following allogeneic (allo) HSCT has reached approximately 30% (4–6), with the majority of mortality being attributed to disease progression. Despite the poor outcomes with allo-HSCT, ALL has been shown to be responsive to immunotherapy, including donor lymphocyte infusion and adoptive T cell therapy (3, 7–10). In fact, ALL is the prototype malignancy for the use of chimeric antigen receptor T cells, highlighting the potential of immunotherapy to eliminate ALL and improve patient outcomes (7–9, 11).

A critical component of immunotherapy development, however, is the identification of tumor antigens against which immunotherapies can be engineered. The paucity of known tumor antigens has been an obstacle to the development of vaccines, antibodies, and cellular immunotherapies. In addition to enabling the specific targeting of the tumor cells, identifying tumor-specific or tumor-associated antigens will minimize the off-target immune toxicities of immunotherapy, including allo-HSCT and cellular therapies. Our group has identified azurophil granule serine proteases, specifically cathepsin G (CG), neutrophil elastase (NE), and proteinase 3 (P3), as immunotherapeutic targets in acute myeloid leukemia (AML) and has developed immunotherapies that target these proteases. Although CD19 has proven to be an effective target in lymphoid malignancies, including ALL (7, 8, 11, 12), there remains a paucity of known immunotherapeutic targets in ALL. However, the response of ALL to targeted immunotherapy highlights the need to further discover ALL-specific/associated antigens. In this report, we focus on CG as a novel antigen for immunotherapy development in ALL.

Cathepsin G is a serine protease largely restricted to the myeloid lineage and is expressed in high levels within azurophil granules in AML blasts and leukemia stem cells and during the promyelocyte stage of neutrophil development. CG is involved in host immunity, cleavage of inflammatory mediators, degradation of extracellular matrix components, antigen presentation, and leukemogenesis (13–16). We previously identified CG as a myeloid leukemia-associated antigen (17, 18). We showed that CG is processed by AML blasts and that a number of human leukocyte antigen (HLA) class I peptides derived from CG were presented by AML. We also demonstrated that cytotoxic T lymphocytes (CTLs) that are expanded to target CG1 (FLLPTGAEA), a HLA-A2-restricted nonameric peptide derived from CG, eliminated AML *in vitro* and *in vivo* (17, 18). Finally, we detected CTLs specific for CG1 in the peripheral blood of AML patients after allo-SCT (17).

Using mass spectrometry, we identified CG1 in the HLA class I-immunoprecipitated fraction from one patient with ALL (18). In addition to our studies, there have been three other reports that suggested CG expression in lymphoid leukemia. CG was reported in chronic lymphocytic leukemia (19) and Hodgkin’s lymphoma (20), and cellular immunity targeting CG eliminated leukemic cells in three patients with ALL (21). These data provided the impetus to further study the immunotherapeutic potential of targeting CG in lymphoid malignancies.

In this study, we demonstrate CG gene and protein expression in ALL cell lines and ALL patient samples. In addition to endogenous expression, we demonstrate that CG can be taken up by ALL. We show that ALL is susceptible to killing by CG1-specific CTLs (CG1-CTLs). Finally, we show that CG expression correlates negatively with ALL patient outcomes.

## Materials and Methods

### Patient Samples and Cell Lines

Patient and healthy donor samples were obtained after appropriate informed consent through an institutional review board approved protocol at the University of Texas MD Anderson Cancer Center (MDACC). Patient, including the samples used in the reverse-phase protein array (RPPA) and UPN1-8, and healthy-donor peripheral blood mononuclear cells (PBMC) and polymorphonuclear lymphocytes (PMN) were isolated from buffy coats after single or double Ficoll gradient centrifugation, respectively, using Histopaque-1077 and Histopaque-1119 (Sigma-Aldrich). SUP-B15 (B lymphoblastic leukemia), SB (B lymphoblast leukemia), RS4;11 (B lymphoblastic leukemia), NALM6 (B lymphoblastic leukemia), Raji (Burkitt’s lymphoma), and T2 (B cell/T cell hybridoma) cell lines were obtained from American Type Culture Collection. Cells were cultured in RPMI 1640 media with 2.5 mM l-glutamine (Hyclone) supplemented with 10% fetal bovine serum, 100 U/mL penicillin, and 100 μg/mL streptavidin (Invitrogen). All cells were cultured at 37°C and 5% CO_2_. Cells lines were validated at the MD Anderson Sequencing and Microarray Facility *via* short tandem repeat DNA fingerprinting and checked for mycoplasma *via* PCR (PromoKine). Raji cells were transduced with HLA-A*0201 as described previously using a lentiviral vector encoding HLA-A*02:01 (18, 22). HLA-A2 expression was verified by flow cytometry prior to using the cell line. HLA-A*0201^+^ Raji cells (Raji-A2) were subsequently used in western blots and cytotoxicity assays, as described.

### RNA Purification and RT-PCR

Purified RNA was extracted via the RNeasy Plus Mini Kit (Qiagen) and used per manufacturer’s instructions. Synthesis of cDNA was performed using the Gene Amp RNA kit (PerkinElmer). The following primer was ordered from Sigma-Aldrich: *Cathepsin G* (forward 5′-AAACACCCAGCAACACATCA-3; reverse 5′-TATCCAGGGCAGGAAACTTG-3′). Actin (forward 5′-CCAGAGCAAGAGAGCTATCC-3′; reverse 5′-CTGTGGTGGTGAAGCTGTAG-3′) served as a loading control. Following denaturation for 5 min at 95°C, samples were amplified for 35 cycles using an iCycler IQ Thermal Cycler (Bio-Rad Laboratories). Samples were run on a 1.5% agarose gel and bands were imaged using GelDoc2000 (Bio-Rad Laboratories) and analyzed by Quantity One software (Bio-Rad Laboratories).

### Cell Lysates and Western Blots

Western blotting for CG was performed as previously described (17). Briefly, cell pellets were suspended in lysis buffer (10 mM/L HEPES [pH 7.9], 10 mM/L KCl, 0.1 mM/L EGTA, 0.1 mM/L EDTA, and 1 mM/L DTT) containing protease inhibitors and underwent freeze–thaw cycles for 15 min to generate whole-cell lysates. Cell lysates were separated on 10% SDS gels by electrophoresis, transferred onto polyvinylidene difluoride membranes, blocked in 5% milk, and stained with anti-CG (Abcam), anti-tubulin (Sigma) antibodies. Blot was rocked in ECL reagent and then imaged using ChemiDoc Touch Imaging System (Bio-Rad).

### CG Uptake by ALL and Normal B Cells

Analysis of the uptake of CG in B-ALL cell lines was carried out by standard flow cytometry methods, as we previously described (23–25). Cells were cocultured in reduced serum medium (0.5% FBS) with irradiated (7,500 cGy) PMN at a ratio of 3:1 (PMN: B-ALL); PMN served as the source for cell-associated CG. After co-incubation, cells were stained with Live/Dead Fixable Ghost Dye (Tonbo), B-ALL markers (Table 1), and IVIG (Privigen) to block the Fc receptor. After surface staining, cells were washed in PBS, fixed in 1% formaldehyde (ThermoFisher Scientific) in PBS and permeabilized in 5% Perm/Wash Buffer (BD). Uptake of CG was determined by staining with anti-CG-FITC (Bio-Rad). Flow cytometry was performed using LSRFortessa Analyzer (BD Bioscience), and analyzed *via* Flowjo software (Treestar).

**Table 1:** Patient characteristics.

Patient	Blast%	Surface markers	Ph^+^	Cytogenetics
UPN1	72	CD10^+^, CD19^+^, CD34^+^, CD20^+^	−	46 XX, −12
UPN2	95	CD10^+^, CD19^+^, CD34^+^, TdT	−	39–47, XX, −6, +5, +8, +11, −18, −19
UPN3	96	CD10^−^, CD19^+^, CD34^+^, CD20^−^	−	46 XX, *t*(4;11)
UPN4	91	CD10^+^, CD19^+^, CD34^dim^, CD20^dim^, TdT	−	47 XY, +21
UPN5	97	CD10^+^, CD19^+^, CD34^+^, CD20^+^	+	Diploid
UPN6	92	CD10^dim^, CD19^+^, CD34^+^, CD20^partial^	−	46 XY
UPN7	87	CD10^+^, CD19^+^, CD34^+^, CD20^−^	+	46 XX, *t*(9;22), +21
UPN8	92	CD10^+^, CD19^+^, CD34^+^, CD20^dim^	−	45, 46 XY, −6, −9

Same methodology was used to determine uptake of CG by normal B cells. After co-incubation with PMN, PBMC were surface stained with lineage antibodies including CD3 (BioLegend), CD14 (BioLegend), CD16 (BioLegend), and CD19 (BD), fixed, permeabilized, and intracellularly stained with anti-CG antibody. B cells were differentiated based on light scatter characteristics as well as being surface CD3^−^/CD14^−^/CD16^−^/CD19^+^. Similar to B-ALL, normal B cells also appear to take up CG (Figure S4 in Supplementary Material).

### CG1-Specific CTLs

To expand CG1-specific CTLs, dendritic cells (DCs) were matured from adherent monocytes and then used as professional APCs (26, 27). Normal PBMC isolated from buffy coats were adhered to a six-well plate at 37°C in Macrophage Serum Free Medium 1× (Gibco). Lymphocytes from the same donor were separated and cocultured with 40 μg/mL of CG1. Cells were then stimulated with interleukin (IL)-7 (10 ng/mL) (rhIl-7, carrier free; BioLegend) and IL-2 (10 ng/mL) (rhIL-2; R&D) over 5 days. Adhered monocytes were matured into monocyte-derived DC through the addition of granulocyte macrophage colony-stimulating factor (GM-CSF) (100 ng/mL; Sanoti), IL-4 (50 ng/mL) (rhIL-4; Tonbo Biosciences), and tumor necrosis factor-α (25 ng/mL; BioLegend). After 5 days, DCs were detached from the six-well plates, cocultured with CG1 peptide (Bio-Synthesis, Inc.) at 40 μg/mL, and combined with the expanded lymphocyte population. Lymphocytes were expanded by coculture with mature DCs and stimulated with IL-7 (10 ng/mL) and IL-2 (25 ng/mL) for an additional 7 days. On day 14 of stimulation, cells were harvested and analyzed *via* flow cytometry by CG1 tetramer staining to determine the percentage of antigen-specific cells that were generated (17). CG1-CTL were identified using the following fluorescently conjugated tetramer and antibodies: PE-CG1/HLA-A*0201 tetramer (Baylor College of Medicine); APC-anti-CD3 (BioLegend); APC/Cy7-anti-CD8 (BioLegend); lineage (Lin) markers including pacific blue-anti-CD4 (BioLegend), CD14 (BioLegend), CD16 (BioLegend), and CD19 (BD) (Figure S1 in Supplementary Material). Prior to coculture with target cells in the cytotoxicity assays, bulk CTLs were enriched using a CD8^+^ T cell isolation MACS kit (Miltenyi Biotec).

### CG1-CTLs Cytotoxicity Assay

A standard cytotoxicity assay was used to determine specific lysis, as previously described (28, 29). T2 cells were cultured overnight with soluble CG (10 μg/mL), washed in RPMI 1640 (HyClone), and resuspended at 1.0 × 10^5^ cells/mL. T2 and target ALL cells were then stained with calcein-AM (Invitrogen) for 15 min at 37°C. Stained cells were then washed three times in RPMI 1640, resuspended at 2.0 × 10^5^ cells/mL, plated onto a Terasaki plate, and cocultured with CG1-specific CTLs at increasing effector:target ratios. After a 4-h incubation period, trypan blue was added to each well to quench fluorescence of dead cells. Fluorescence was measured using a Cytation 3 Imaging Reader (BioTek). Percent specific lysis was calculated by using the following formula:

$$\left(1-[{\text{fluorescence}}_{\text{target}+\text{effector}}-{\text{fluorescence}}_{\text{media}}]/[{\text{fluorescence}}_{\text{target alone}}-{\text{fluorescence}}_{\text{media}}]\right)\times 100.$$

T2 cells pulsed with CG1 and irrelevant E75 peptide were used as positive and negative controls, respectively.

The HLA typing of patient samples UPN5 and UPN7 was performed at the MD Anderson HLA-typing laboratory and was obtained from the patients’ medical records. HLA typing of UPN2 was not included in the patient’s medical record; therefore, typing was performed by staining cells with anti-HLA-A*0201 antibody (clone BB7.2, BD Bioscience), as previously described (17, 30). To show the effects of CG uptake on cytotoxicity, Raji-A2 cells were cocultured with purified CG protein (Athens) for 24 h prior to performing the cytotoxicity assay.

### Samples and Sample Preparation for the RPPA Analysis

Peripheral blood and bone marrow (BM) specimens were collected from 130 patients with newly diagnosed ALL, and healthy donors evaluated at MDACC from 1992 to 2007. A paired relapse sample was also collected when available. Of the 130 patients, all were treated at MDACC and were evaluable. Among the treated patients, all received regimens that contained hyper-CVAD, with 23 also receiving rituximab, 9 with Philadelphia chromosome (Ph)-positive disease also received a tyrosine kinase inhibitor, and 1 Ph-positive case was treated with hyper-CVAD in combination with both rituximab and imatinib.

For RPPA, fresh samples were collected and enriched for leukemic cells using Ficoll-Hypaque (Sigma-Aldrich) density-gradient separation to yield a mononuclear fraction. The blast purity in the samples reached approximately 98% as determined by flow cytometry. Whole-cell lysates were prepared from samples that were normalized to a concentration of 1 × 10^4^ cells/μL. RPPA was carried out as previously described (31, 32). Briefly, five serial dilutions of patient samples were printed onto slides along with normalization and expression controls. Slides were stained with 232 strictly validated primary antibodies, including one against CG (Abcam ab8816, Cambridge, MA, USA). The stained slides were analyzed using MicroVigene software (Vigene Tech, Carlisle, MA, USA) to produce quantified data.

### Statistical Analysis

The associations between CG expression and categorical clinical factors were evaluated in R using standard *t*-tests, linear regression, and mixed-effects linear models. OS was measured from the date of diagnosis to the date of death due to any cause, and survival curves were generated using the Kaplan–Meier method. In addition, a Cox proportional hazards regression model was constructed to investigate the association between CG protein levels and OS. All statistical analyses for clinical outcome were computed using the Statistica software V.12 (StatSoft, Tulsa, OK, USA).

## Results

### ALL Patient Samples and Cell Lines Demonstrate Variable Expression of CG Transcript and Protein

**Figure 1:**
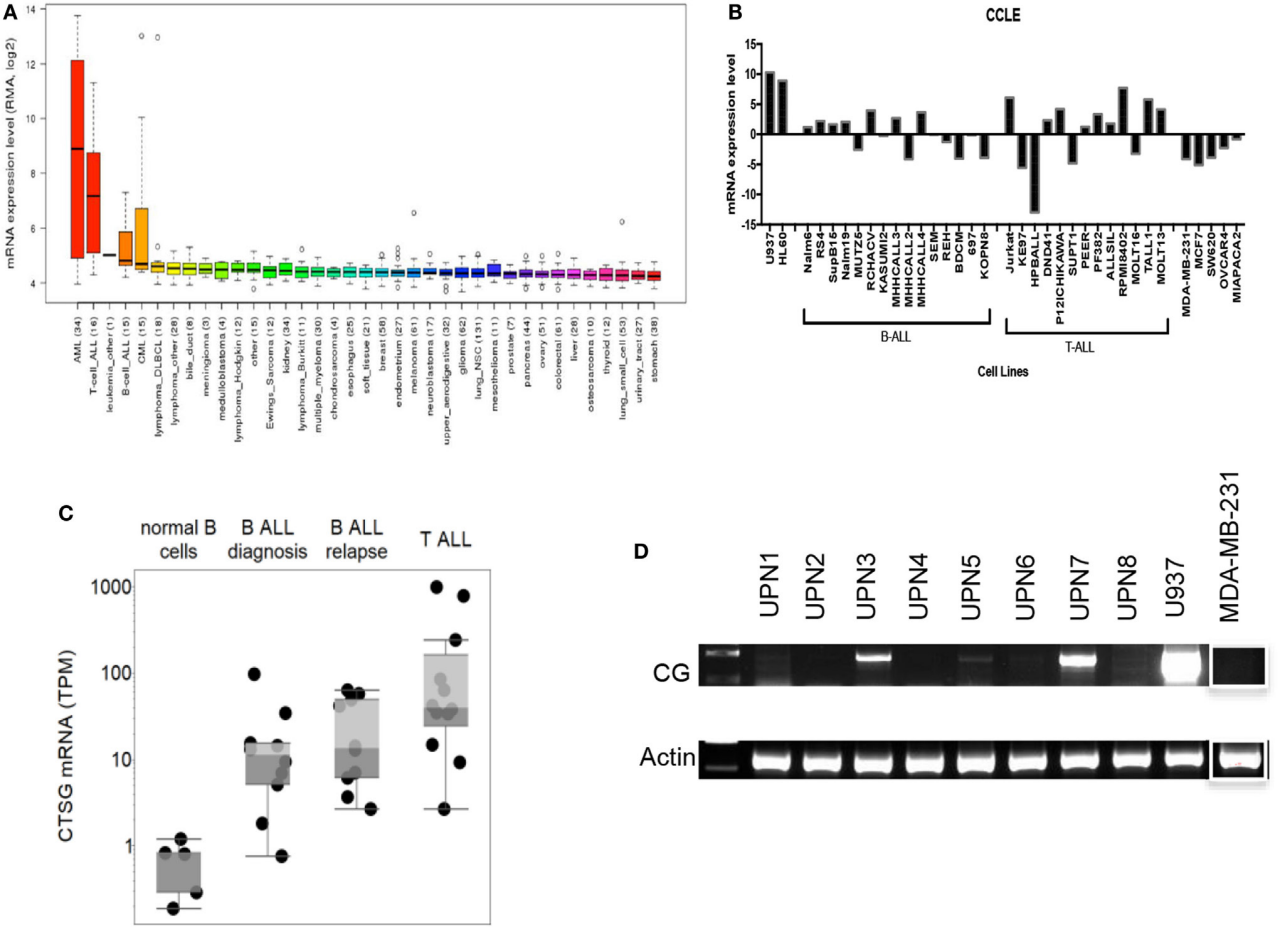
Cathepsin G (CG) transcript is detected in B-ALL patient samples and cell lines. **(A)** According to Cancer Cell Line Encyclopedia (CCLE), ALL cell lines express CG transcript at higher levels compared with other malignancies. High-expressing acute myeloid leukemia (AML) cell lines are shown for comparison. **(B)** CG mRNA expression is not ubiquitous to all ALL. CG levels are lower in B-ALL and T-ALL cell lines compared with U937 and HL60 AML cell lines, but are higher compared with solid tumors; MDA-MB-231 (breast cancer), MCF7 (breast cancer), SW620 (colorectal adenocarcinoma), OVCAR-3 (ovarian adenocarcinoma), and MIA PaCa-2 (pancreatic carcinoma). **(C)** The Cancer Genome Atlas shows higher CG expression at the transcript level in B-ALL compared to normal B cells. Additionally, mRNA expression is higher in relapsed B-ALL compared with B-ALL at time of diagnosis. T-ALL has the highest CG expression at the transcript level. **(D)** RT-PCR of patient B-ALL shows prominent CG expression in three B-ALL patient samples (UPN3, UPN5, and UPN7; Table 1). AML cell line U937 and breast cancer cell line MDA-MB-231 were used as positive and negative controls, respectively. Actin was used as a loading control. The white boxes outlining the MDA-MB-231 bands indicate that they were transposed from a different region of the same gel.

We first employed the Cancer Cell Line Encyclopedia (CCLE) database to investigate CG transcript expression by malignant tissues. CCLE data demonstrate a higher expression of CG transcript in B-ALL compared with other malignancies, although as predicted, the expression is lower than that observed in AML (Figure 1A). As predicted, CCLE also confirms higher expression of CG by B and T cell ALL cell lines, compared with other tumor cell lines (Figure 1B; Table S1 in Supplementary Material). Furthermore, according to The Cancer Genome Atlas, the expression of CG mRNA is higher in relapsed B-ALL compared to B-ALL at the time of diagnosis. Also, T-ALL CG mRNA expression is greater than that seen in B-ALL and normal B cells (Figure 1C; Table S2 in Supplementary Material). This implicates CG as a possible marker of more aggressive ALL. We also confirm variable expression of CG transcript by RT-PCR using eight B-ALL patient samples and demonstrate the absence of CG transcript in some of the patient samples (Figure 1D; Table 1).

**Figure 2:**
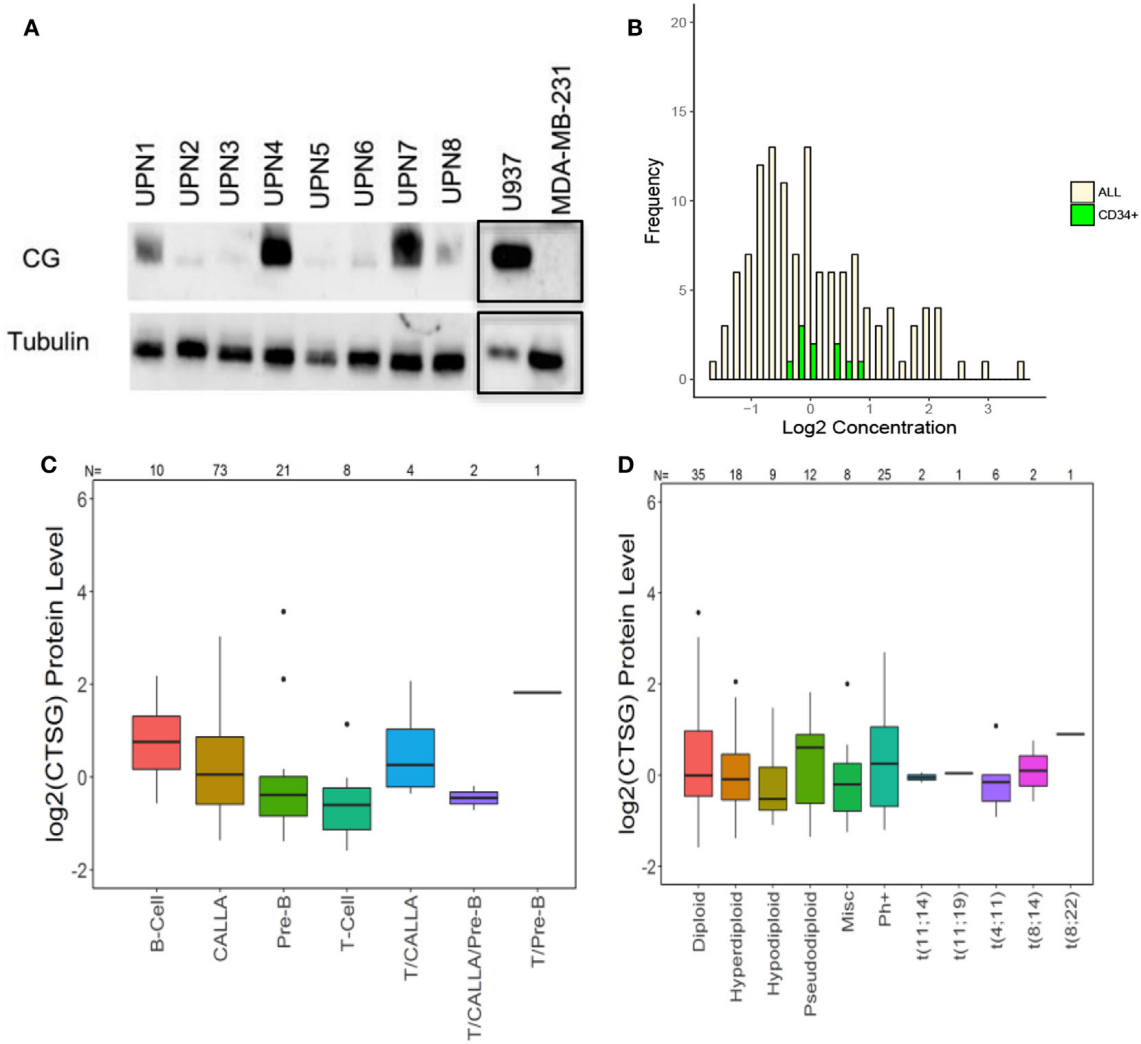
Cathepsin G (CG) is expressed at the protein level in B-ALL patient samples. **(A)** Western blots demonstrate CG protein in whole-cell lysates from eight different B-ALL patient samples (Table 1). Gels were loaded with 30 μg of protein. U937 and MDA-MB-231 cell lines were used as positive and negative controls, respectively. Tubulin was used as a loading control. The black boxes outlining the U937 and MDA-MB-231 bands indicate that they were transposed from a different gel that was run in parallel. **(B)** Reverse-phase protein array was used to quantify protein levels of CG in blasts from 130 newly diagnosed ALL patients (yellow bars). Controls included healthy donor CD34^+^ progenitor cells (green bars). **(C)** Box plots show the levels of CG expression in ALL subtypes according to the French–American–British classification. **(D)** The relationship of CG protein levels is demonstrated with both normal and aberrant cytogenetic subgroups across 130 patient samples. Abbreviations: Misc, miscellaneous.

We then used western blot analysis and RPPA to determine the expression of CG by ALL at the protein level. Western blots show variable expression of CG in the ALL samples, although CG was detected in all eight patient samples (Figure 2A). Furthermore, along with demonstrating variable expression across patient samples, RPPA confirms that CG protein levels are increased in ALL blasts as compared to normal CD34^+^ hematopoietic cells (Figure 2B). Although CG protein levels may seem lower in a sizeable proportion of ALL blasts compared with normal CD34^+^ cells, the mean value of CG in ALL cells was 0.138 while the mean value of CG in CD34^+^ cells was 0.122. Homoskedastic *t*-tests were performed assuming equal variance as well as unequal variance, which produced *P*-values of 0.96 and 0.92, respectively. This provides strong evidence that the means are not different. In addition, RPPA analysis demonstrates protein-level expression of CG across various ALL subtypes (Figure 2C), as well as differing levels of CG expression in normal and aberrant cytogenetic subgroups (Figure 2D).

### B-ALL Can Take Up Exogenous CG

**Figure 3:**
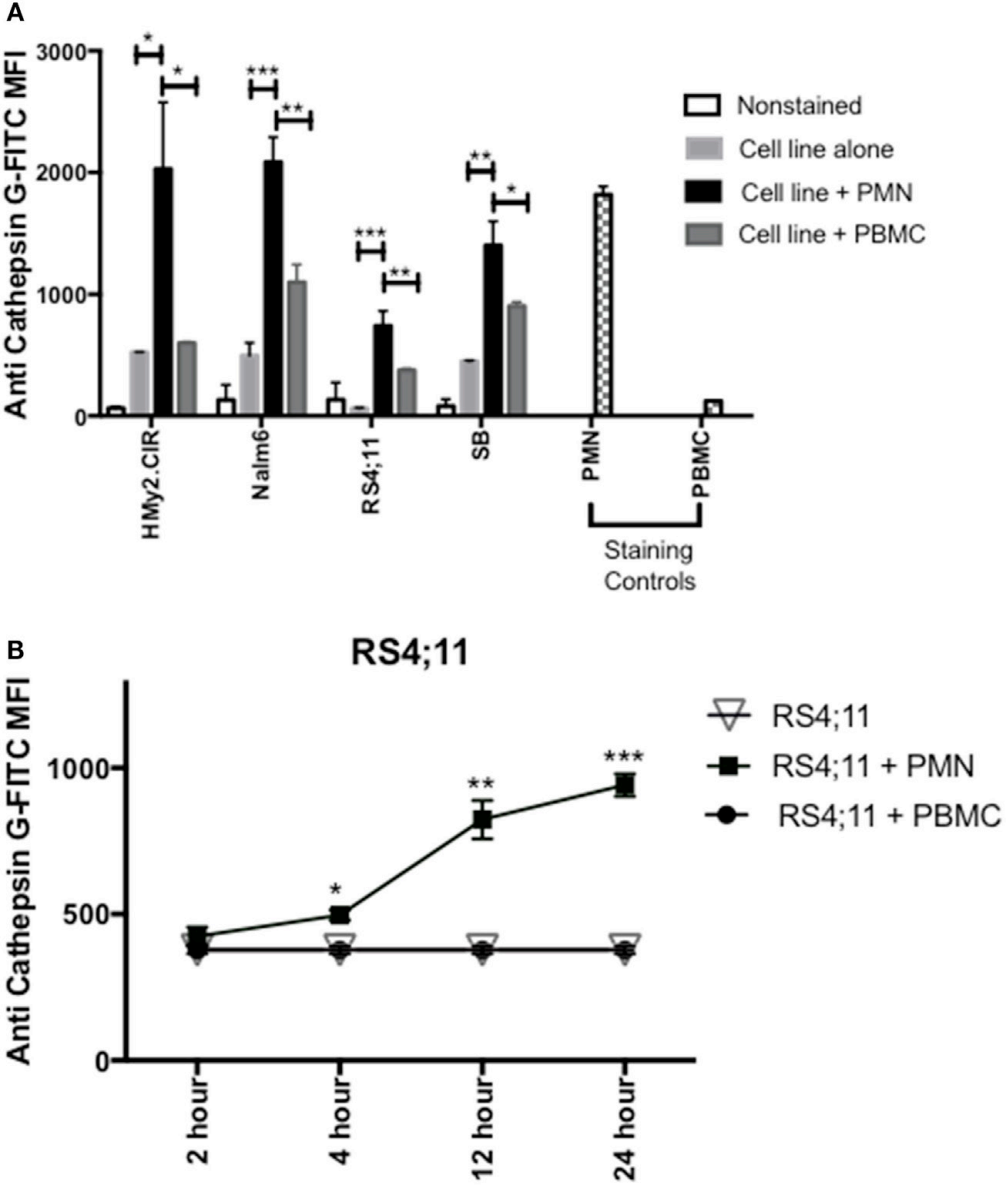
PMN-associated Cathepsin G (CG) is taken up by B-ALL cell lines. **(A)** Flow cytometry detected intracellular CG in B-ALL cell lines after coculture with whole PMN at a ratio of 3:1 for 12 h. Cells were intracellularly stained with anti-CG antibody. PMN and peripheral blood mononuclear cells (PBMC) were used as positive and negative staining controls, respectively. *P < 0.05; **P < 0.01; ***P < 0.001. **(B)** RS4;11 cells were cocultured with whole PMN or PBMC at a ratio of 3:1 for 2, 4, 12, and 24 h and analyzed by flow cytometry for intracellular uptake of CG using anti-CG antibody. Cells cultured in the presence or absence of PBMC were used as negative controls. PMN and PBMC were used as positive and negative staining controls, respectively. Flow cytometry indicated that uptake of extracellular CG occurs in HMY2.CIR, Nalm6, and RS4;11 cell lines. Graphs display the mean ± SEM fold increase in median fluorescence intensity (MFI). Data represent triplicate wells from four independent experiments. *P < 0.05; **P < 0.01; ***P < 0.001.

Since previous studies (33, 34), including our published data (23–25, 30), have shown that azurophil granules serine proteases, including CG, P3, and NE, can be taken up by tumor cells and since we note variable and at times absent expression of CG in some ALL samples, we next examined whether CG can be taken up by ALL. To differentiate CG uptake from endogenous expression, we selected four ALL cell lines, HMy2.CIR, RS4, SB, and Nalm6, that have low endogenous CG expression (Figure 3A). Cells were cocultured overnight with whole PMN, as the source for CG, or PBMC, which have lower expression of CG, at a ratio of 3:1 (PMN:ALL) (Figure 3A). Flow cytometry analysis of intracellular CG staining using a gating strategy that included the ALL cell lines and excluded PMN based on forward and side-scatter properties and CD16 negativity (Table S3 and Figure S2 in Supplementary Material) indicates significant uptake of PMN-associated CG by HMy2.CIR, Nalm6, RS4, and SB B-ALL cell lines (Figure 3A). We also detected an increase in intracellular CG after coculture of ALL with PBMC, although at a much lower level than what we observed with PMN. We attribute this to the CG that is known to be expressed by monocytes, which are found within the PBMC (16, 35). Further, the degree of uptake differs among the cells lines. Uptake was time-dependent, and increases with the longer durations of coculturing with PMN (Figure 3B). Negative controls included RS4 cell line alone or cocultured with irradiated PBMC. PMN and PBMC were used as positive and negative staining controls, respectively.

Furthermore, our data suggest uptake of CG by primary ALL. Specifically, we detected absence of CG transcript in UPN2 and UPN4 (Figure 2D) but detected CG protein in these patient samples (Figure 3A). Together, the data show that B-ALL cell lines lack endogenous CG but can internalize CG from exogenous sources.

### B-ALL Is Susceptible to Killing by CG1-CTLs

**Figure 4:**
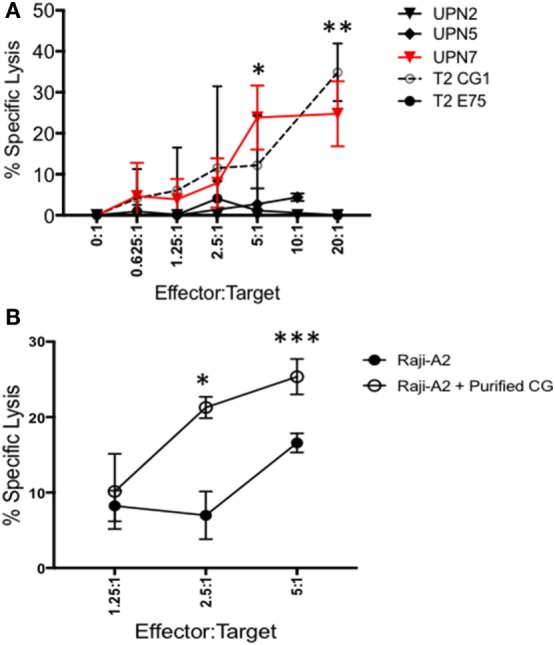
ALL shows susceptibility to killing by CG1-cytotoxic T lymphocytes (CTLs). Patient samples (UPN2, UPN5 and UPN7), T2 cells, and Raji-A2 cells were loaded with calcein-AM, and then cocultured with CG1-CTLs for 4 h at various E (effector): T (target) ratios. Cytotoxicity was determined by measurement of intracellular calcein-AM in live cells. T2 cell lines were pulsed with CG1 and E75 peptide as positive and negative controls, respectively. **(A)** UPN7, which endogenously expresses Cathepsin G (CG), and **(B)** Raji-A2 pulsed with purified CG protein (see Figure S5 in Supplementary Material) showed increased killing by CG1-CTL. Cytotoxicity data are means ± SEM from triplicate wells from a representative experiment. *P < 0.05; **P < 0.01; ***P < 0.001.

Because CG1 has been effectively targeted in myeloid leukemia using CG1-CTLs (17, 18), we investigated whether CG1-CTLs can kill ALL. Based on our data showing that patient sample UPN7 (HLA-A*0201-positive) endogenously expresses CG at the transcript and protein levels (Figures 1D and 2A), UPN7 ALL patient sample was cocultured with CG1-CTLs for 4 h using a standard calcein-AM cytotoxicity assay (28, 29). T2 cells were pulsed with CG1 or E75 (HER2-derived HLA-A2-restricted peptide) (27) as positive and negative controls, respectively. Data indicate a dose-dependent killing of UPN7 by CG1-CTLs (Figure 4A). The percent specific lysis reaches a maximum of approximately 30% at higher doses of CTLs and killing is observed at the lowest ratio of 0.625:1 (effector:target) (Figure 4A). T2 cells pulsed with CG1 also show dose-dependent killing by CG1-CTLs (positive killing control), whereas T2 cells pulsed with E75 demonstrate no significant target lysis (negative control). Conversely, no target killing of two HLA-A*0201 negative samples (UPN2 and UPN5) was detected, despite low expression of CG protein by these samples. The HLA typing of the samples used in the cytotoxicity assays is shown in Table S4 and Figure S3 in Supplementary Material. Furthermore, we cultured Raji-A2 cells with purified CG protein (Figure S5 in Supplementary Material) and confirmed increased killing of Raji-A2 after coculture with CG1-CTLs (Figure 4B).

### CG Expression Is Associated with an Aggressive ALL Phenotype

**Figure 5:**
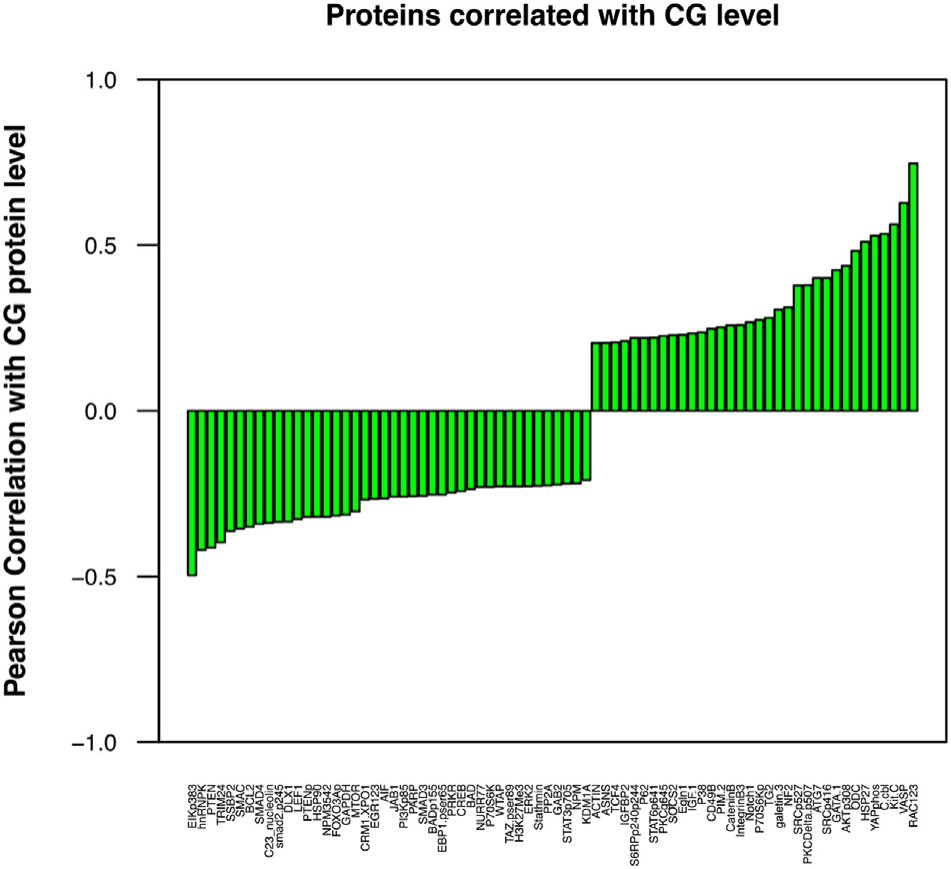
Cathepsin G (CG) protein expression correlates with the concurrent expression of known leukemia-associated proteins. Pearson correlation coefficient analysis was performed to determine the association between the levels of CG and various selected proteins that are known to contribute to leukemogenesis and progression of ALL. These proteins were identified with a correlation coefficient threshold of 0.2. These proteins were assessed for both co-occurrence and inverse correlation with CG1; cell adhesion molecules TG2 and ITGB3, oncogene C-kit, proliferation-associated protein GATA1, and vascular protein VASP were among the proteins expressed at highest levels in association with CG, while tumor suppressor protein PTEN, apoptotic proteins SMAC and BCL2, and differentiation-promoting protein SSBP2 were expressed at low levels. All proteins were identified with a correlation coefficient threshold of 0.2.

Although a direct correlation between protein expression by leukemic cells and tumor behavior may not be readily apparent, individual proteins may affect the aggressive phenotype of a malignant cell indirectly through interactions with other cellular proteins. We have correlated the expression of a number of proteins (36–38), including CG (18), and AML aggressiveness. A Pearson correlation coefficient analysis was, therefore, performed in this ALL patient cohort to determine the association between the levels of CG and these proteins. Figure 5 demonstrates a similar correlation between CG protein expression and the expression of many proteins that have known associations with tumor behavior and response to therapy. Among these, CG-expressing tumor cells were found to concurrently express high levels of adhesion proteins, such as TG2, ITGB3, IGFBP2, IGF1, CD49B, and Galectin 3. Lower expression of stem cell regulating proteins NUR77 and TCF4 and differentiation-promoting proteins SSBP2, along with higher expression of Gata1, Pim2, and Notch 1 were positively associated with CG levels, suggest a loss of stem cell regulation and alteration in differentiation. Increased Hippo pathway regulation *via* increased NF2, and inactivation of YAP by phosphorylation is suggested; however, decreased phosphorylation of TAZ is also observed. CG expression is negatively correlated with apoptosis regulating proteins Smac, BCL2, AIF, and Bad. CG expression is also associated with increased signal transduction and proliferation, evidenced by higher levels of phosphorylated (activated) AKT, PKCδ, SRC, S6RP, and P70S6K, while Stat pathways activity seems to be downregulated as CG is negatively correlated with phosphorylation of Stat3 and 6. Likewise, CG expression is inversely correlated with TGFβ-Smad signaling as levels of pSmad2, Smad3, and Smad4. An increase in pro-vascularization is suggested by a positive association with hypoxia-associated/vascular proteins, such as VASP and EGLN1. Histone methylation/acetylation proteins (hnRNPK, WTAP, H3k4me3, KDM1A) are found to be expressed at lower levels in the presence of CG protein. The actual consequences of modulated CG on each of these pathways require further experimental validation.

**Figure 6:**
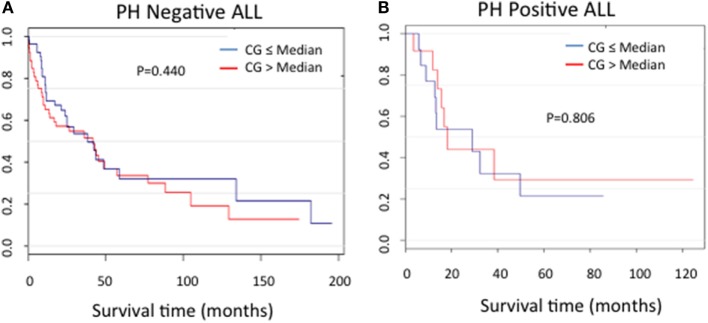
Cathepsin G (CG) protein expression and ALL survival. Kaplan–Meier curves show no significant differences in survival in patients with **(A)** Ph-negative (*n* = 104; *P* = 0.44) or **(B)** Ph-positive (*n* = 25; *P* = 0.806) ALL according to CG expression.

In order to assess the correlation between CG expression and clinical outcomes, the ALL patients used in RPPA for whom survival information was available were categorized into Ph-positive (*n* = 25) and Ph-negative (*n* = 104) subgroups. For both patient cohorts, no difference in the duration of survival was noted between patients in the context of CG expression (*P* = 0.440 for Ph-negative ALL; *P* = 0.806 for Ph-positive ALL) (Figures 6A,B).

## Discussion

In this manuscript, we demonstrate the expression of the myeloid azurophil granule protease CG by ALL. Specifically, we demonstrate CG expression by B-ALL and T-ALL at the transcript and protein levels. Expression is demonstrated in cell lines and patient samples, and correlates with poor prognosis. In addition to endogenous expression, we show time-dependent uptake of PMN-associated CG by B-ALL. Finally, we demonstrate that patient ALL can be targeted effectively by CG1-CTLs.

Cathepsin G is known to be predominantly expressed by normal myeloid progenitor cells and also in primary AML blasts and leukemic stem cells (39, 40). Our study demonstrates the expression of the myeloid serine protease CG by B- and T-ALL, in cell lines as well as patient samples. This expression may be due to endogenous expression, as is shown in some patient samples and cell lines (Figure 1), and is also secondary to CG uptake (Figure 3). It may be noteworthy that our analysis not only identifies higher CG expression in B-ALL cells as compared to normal B-cells, but also establishes a hierarchy of expression, wherein CG expression at the transcript level is higher in T-ALL and relapsed B-ALL. The data, therefore, implicate CG as a possible marker of poor prognosis in ALL, similar to what we reported in AML (18), and also validate the uptake of serine proteases as a shared mechanism among tumors.

Furthermore, protein localization within the tumor cell can facilitate peptide presentation by surface class I HLA (24, 41). We have shown that CG, NE, and P3 are aberrantly expressed in AML blasts outside azurophil granules and that the aberrant expression may augment their presentation by AML (17, 24). Since ALL lacks azurophil granules, which normally house serine proteases, ALL may efficiently present CG due to its aberrant localization, irrespective of uptake or endogenous expression. Furthermore, the lack of azurophil granules in ALL could explain the absence of CG protein expression in some patient samples where CG expression at the mRNA level was detected (Figures 1 and 2). Specifically, azurophil granules shield proteases, including CG, from intracellular degradation. The absence of these granules could, therefore, facilitate intracellular degradation of serine proteases, including CG (24). In addition to protein expression, we have previously eluted the HLA-A2 restricted peptide CG1 from the surface of ALL. Cytotoxicity data (Figure 4) along with a prior report by Fujiwara et al. showing T cell responses to CG in three patients with ALL point to the potential relevance of CG as an immunotherapeutic target in ALL (21). These data showing that CG expression is not limited to the myeloid lineage and is presented by HLA class I on ALL cell surface warrant further investigation of CG- and CG1-targeting immunotherapies in lymphoid malignancies.

Moreover, we have previously reported that a number of tumor types, including B cell-derived malignancies, take up and cross-present the serine proteases NE and P3, and are consequently rendered susceptible to killing by CTLs targeting the HLA-A2-restricted peptide PR1 (23, 24, 30). Cross-presentation is a well-defined immunologic mechanism whereby exogenous antigens are taken up and cross-presented by HLA class I molecules on the cell surface (42). We now extend these findings to CG. Although other investigators have shown that CG, in addition to NE, can be taken up by lung cancer (34), this is the first report showing CG uptake by a hematologic malignancy. This observation is highly relevant since ALL originates in the BM, where CG expression is higher than that found in other tissues. The uptake and cross-presentation of CG by ALL may be a mechanism that renders ALL susceptible to CG-targeting immunotherapy, thereby expanding the repertoire of ALL-associated immunotherapeutic targets. Nevertheless, we recognize that a significant proportion of ALL lacks CG and, therefore, would not be susceptible to CG-targeting immunotherapy. Furthermore, the ability of ALL to take up and cross-present CG from the BM microenvironment suggests a more widespread process by which antigens that are endogenously absent in ALL can be taken up by the malignant cells, thereby leading to a much broader arsenal of ALL targets.

These data also point to tissue-specificity of CG uptake, since we have shown that breast cancer cells do not take up CG (25), which could be due to a shared receptor between distinct tumor types that facilitates the uptake of serine proteases (43). Our results also indicate that the efficiency and rate of CG uptake varies between different cell lines. Furthermore, previous studies have cited time variations for antigen cross-presentation, with DC cross-presentation occurring as early as 4 h (24, 44), while cross-presentation by solid tumor cells occurred at later time points (24 h) (23, 30). Considering the role of B-lymphocytes as professional APCs, we expect cross-presentation by ALL to occur at early time points, as we have shown that cross-presentation of NE and P3 by the B-ALL cell line HMy2.CIR occurred as early as 6 h. Since priming an immune response requires interactions between co-stimulatory molecules and APCs and their cognate receptors on T cells, our data presented here and published reports suggest that ALL can prime an immune response against CG-derived peptides; however, we recognize that we do not provide data to directly support this hypothesis. Nevertheless, the data presented here highlight the role of CG1 as a target in ALL.

Similar to the results obtained with cell lines, our data show that all B-ALL patient samples express CG to varying extents (Figures 1 and 2). Three patient samples (UPN 3, 5, and 7) exhibited endogenous expression of CG at the transcript level (Figure 1D). However, western blot analysis shows some degree of expression by all eight patient samples. The discrepancy between transcript and protein expression suggests that CG uptake may play a major role in regulating CG expression by ALL and may prognosticate for poor outcomes in ALL. The poor prognostic role of CG in ALL is not surprising, given that previous studies have correlated higher CG levels with aggressive behavior in breast carcinoma (45) and inferior survival in AML (18). Furthermore, two prior reports have correlated the uptake of serine proteases by lung and breast cancer cells with increased tumor cell proliferation (33, 34). Although the mechanistic studies to explain these observations are scarce, CG has been reported to induce the adhesive action of E-cadherin between tumor cells (46), which may precipitate tumor invasiveness due to E-cadherin-mediated collective metastasis (47) or apoptosis inhibition (48). Furthermore, CG also has been shown paradoxically to suppress monocyte activation, which may contribute additionally to tumor aggressiveness as a result of a downregulated immune response (49). We have shown that CG expression correlated negatively with survival in AML, especially in the presence of intermediate cytogenetics and *FLT3* mutations (18). Similarly, a high level of CG expression is shown to be associated with a higher risk of relapse in ALL (Figure 1C), which may be attributed to the associations demonstrated between CG and several other proteins potentially involved in leukemogenesis. The concurrent expression of cell adhesion molecules and oncogenes with CG is shared between AML and ALL cells and has been ascribed to CG-mediated modulation and cleavage; in addition to the phosphorylation of oncogenes, such as SRC, YAP1, and FOXO3, in ALL, this supports their role in the association between CG expression and worse prognosis. Furthermore, higher levels of proliferation-associated proteins in conjunction with lower levels of apoptotic proteins, as observed in CG-expressing cells, is likely to encourage the uncontrolled growth of leukemic cells in the pathogenesis of ALL. While vascular proteins have not been directly implicated in the progression of ALL, EGLN1 is known to negatively regulate hypoxia-inducible factor-1-alpha (HIF-1) and induce a non-hematological malignancy (50). Given the potential of HIF-1 to act as a tumor suppressor in AML (51), a similar effect in ALL may justify its contribution to leukemogenesis. Nevertheless, the observation that higher CG expression correlates with ALL relapse highlights the potential for CG1-CTLs therapy in this patient cohort.

In conclusion, these data demonstrate the expression of a myeloid tumor antigen by ALL. Moreover, our results further highlight the role of cross-presented antigens as targets for immunotherapy. Specifically, our findings expand on the potential to target CG with immunotherapy in ALL.

## Author Contributions

MK, SC, and PS performed experiments, analyzed data, and wrote the manuscript. JR performed bioinformatics analyses and wrote parts of the manuscripts. AP, AAP, CK, NQ, MZhang, MZope, JG, MQ, and HJ performed experiments, analyzed data, and assisted with manuscript editing. LJ and KC-D assisted with flow cytometry analyses and edited the manuscript. YQ and SK performed RPPA experiments, analyzed data, and wrote the manuscript. EM and JM provided major contributions to the study design and experimental planning. GA was the principal investigator of this study, designed experiments, analyzed data, and wrote the manuscript and the revisions. MZhang performed experiments, analyzed data, and assisted with manuscript editing.

## Conflict of Interest Statement

The authors declare that the research was conducted in the absence of any commercial or financial relationships that could be construed as a potential conflict of interest.

## Supplementary Material

Figure S1 Gating strategy used to determine CG1-CTL frequency following expansion. Cytotoxic T lymphocytes (CTLs) were stained with CG1/human leukocyte antigen (HLA)-A*0201 tetramer in addition to CD3, CD8, lineage (lin) markers CD4, 14, 16, 19, and live/dead Ghost Violet stain. Frequencies of CG1-CTL (CG1/HLA-A*0201 tetramer^+^) is determined from live, lin^−^, CD3^+^, CD8^+^ cell populations.

Figure S2 Gating strategy used to determine uptake of cathepsin G (CG) by ALL cell lines. NALM6 cells were surface stained with antibodies including CD10 (BioLegend), CD16 (BioLegend), CD19 (BD), and CD38 (BioLegend). Cells were fixed, permeabilized and intracellularly stained with anti-CG antibody. B-ALL cells were differentiated based on light scatter characteristics as well as established surface phenotype (CD10^+^/CD16^−^/CD19^+^/CD38^+^).

Figure S3 Human leukocyte antigen (HLA)-A*0201 status of UPN2. UPN2 ALL was stained with anti-HLA-A*0201 antibody (clone BB7.2) and analyzed using flow cytometry. Data demonstrate UNP2 to be HLA-A2*0201 negative. The HLA-A*0201-positive cell line, T2, was used as a positive control.

Figure S4 PMN-associated cathepsin G (CG) is taken up by normal B cells. Flow cytometry detected intracellular CG in the B cell population from normal donor peripheral blood mononuclear cells (PBMC) that were cocultured with irradiated whole PMN at a ratio of 3:1 overnight. PBMC were surface stained with lineage antibodies, including CD3, CD14, CD16, and CD19, and intracellularly stained with anti-CG antibody. B cells were identified based on light scatter characteristics as well as being surface CD3^−^/CD14^−^/CD16^−^/CD19^+^. Median fluorescence intensity (MFI) shown represent CG expression within the gated B-cell population. Non-stained and stained normal PMN were used as negative and positive staining controls, respectively. **P* < 0.05.

Figure S5 Raji-A2 cells take up exogenous cathepsin G (CG). Raji-A2 cells were cultured with purified CG (10 μg/mL) for 24 h. Western blot analysis shows uptake of CG by Raji-A2. Western blots demonstrate CG protein in whole-cell lysates from Raji-A2. Gels were loaded with 30 μg of protein. U937 cell line was used as a positive control. Actin was used as a loading control.
