# Supplementary figures and images for: Cathepsin G Is Expressed by Acute Lymphoblastic Leukemia and Is a Potential Immunotherapeutic Target
Source: Front Immunol. 2018 Jan 25;8:1975. doi: 10.3389/fimmu.2017.01975 (PMC5790053; doi:10.3389/fimmu.2017.01975)

# Supplementary Figure 1.

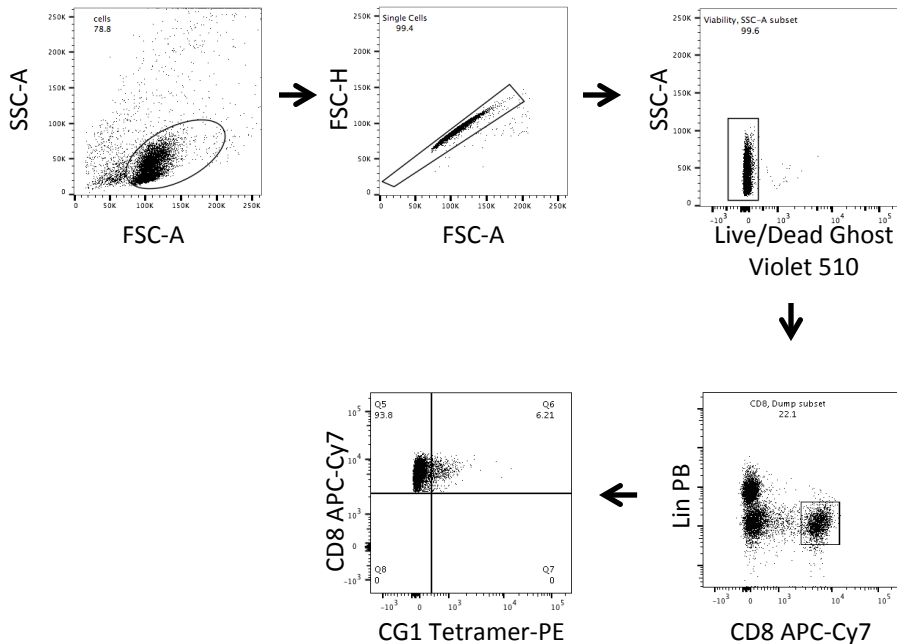

Supplement: Figure S1 — Gating strategy used to determine CG1-CTL frequency following expansion. Cytotoxic T lymphocytes (CTLs) were stained with CG1/human leukocyte antigen (HLA)-A*0201 tetramer in addition to CD3, CD8, lineage (lin) markers CD4, 14, 16, 19, and live/dead Ghost Violet stain. Frequencies of CG1-CTL (CG1/HLA-A*0201 tetramer+) is determined from live, lin−, CD3+, CD8+ cell populations. [file Image_1.PDF]

## Supplementary Figure 2.

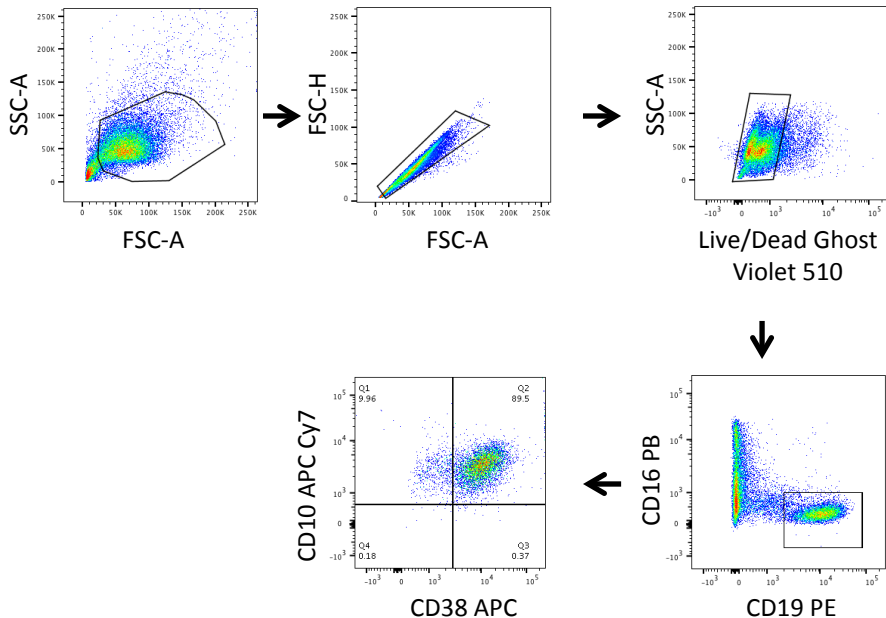

Supplement: Figure S2 — Gating strategy used to determine uptake of cathepsin G (CG) by ALL cell lines. NALM6 cells were surface stained with antibodies including CD10 (BioLegend), CD16 (BioLegend), CD19 (BD), and CD38 (BioLegend). Cells were fixed, permeabilized and intracellularly stained with anti-CG antibody. B-ALL cells were differentiated based on light scatter characteristics as well as established surface phenotype (CD10+/CD16−/CD19+/CD38+). [file Image_2.PDF]

# Supplementary Figure 3.

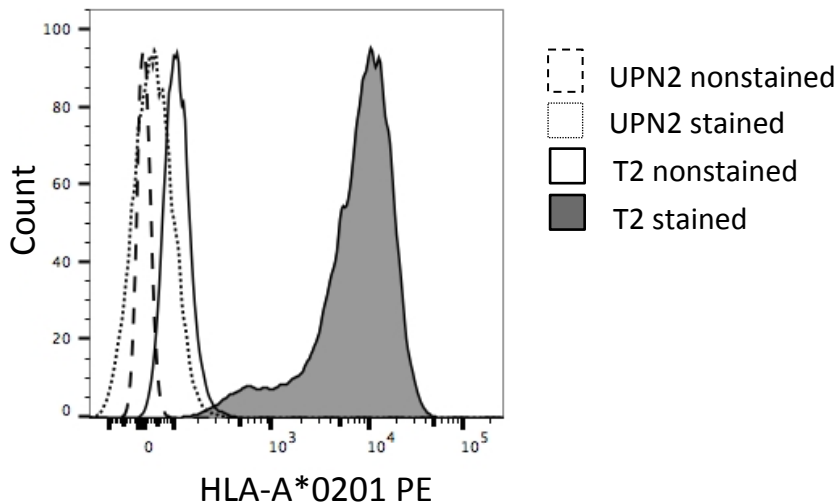

Supplement: Figure S3 — Human leukocyte antigen (HLA)-A*0201 status of UPN2. UPN2 ALL was stained with anti-HLA-A*0201 antibody (clone BB7.2) and analyzed using flow cytometry. Data demonstrate UNP2 to be HLA-A2*0201 negative. The HLA-A*0201-positive cell line, T2, was used as a positive control. [file Image_3.PDF]

Supplementary Figure 4.

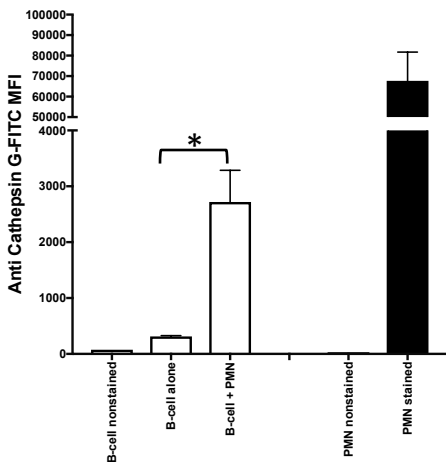

Supplement: Figure S4 — PMN-associated cathepsin G (CG) is taken up by normal B cells. Flow cytometry detected intracellular CG in the B cell population from normal donor peripheral blood mononuclear cells (PBMC) that were cocultured with irradiated whole PMN at a ratio of 3:1 overnight. PBMC were surface stained with lineage antibodies, including CD3, CD14, CD16, and CD19, and intracellularly stained with anti-CG antibody. B cells were identified based on light scatter characteristics as well as being surface CD3−/CD14−/CD16−/CD19+. Median fluorescence intensity (MFI) shown represent CG expression within the gated B-cell population. Non-stained and stained normal PMN were used as negative and positive staining controls, respectively. *P < 0.05. [file Image_4.PDF]

# Supplementary Figure 5

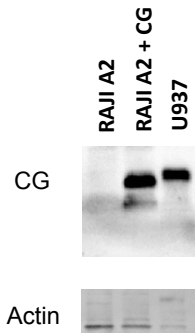

Supplement: Figure S5 — Raji-A2 cells take up exogenous cathepsin G (CG). Raji-A2 cells were cultured with purified CG (10 μg/mL) for 24 h. Western blot analysis shows uptake of CG by Raji-A2. Western blots demonstrate CG protein in whole-cell lysates from Raji-A2. Gels were loaded with 30 μg of protein. U937 cell line was used as a positive control. Actin was used as a loading control. [file Image_5.PDF]
