# Supplementary material for: Cathepsin G Is Expressed by Acute Lymphoblastic Leukemia and Is a Potential Immunotherapeutic Target
Source: Front Immunol. 2018 Jan 25;8:1975. doi: 10.3389/fimmu.2017.01975 (PMC5790053; doi:10.3389/fimmu.2017.01975)
Supplement: Supplementary file 7 [file Table_2.docx]

**Supplementary Table 2.** Kruskal-Wallis test (*P*= 0.000955) followed by posthoc Kruskal – Nemenyi pairwise test (P values are included in the table).

|  | **B-ALL Diagnosis** | **B-ALL Relapse** | **Normal B cells** |
| --- | --- | --- | --- |
| **B-ALL Relapse** | 0.91891 |  |  |
| **Normal B cells** | 0.11609 | **0.03042** |  |
| **T-ALL** | 0.15535 | 0.48422 | **0.00041** |
