# Supplementary material for: Cathepsin G Is Expressed by Acute Lymphoblastic Leukemia and Is a Potential Immunotherapeutic Target
Source: Front Immunol. 2018 Jan 25;8:1975. doi: 10.3389/fimmu.2017.01975 (PMC5790053; doi:10.3389/fimmu.2017.01975)
Supplement: Supplementary file 8 [file Table_3.docx]

| **Cell line** | **CD10** | **CD19** | **CD34** | **CD38** |
| --- | --- | --- | --- | --- |
| **RS4;11** | - | + | - | + |
| **SB** | - | + | - | + |
| **SUPB15** | + | + | + | + |
| **NALM6** | + | + | - | + |
| **HMy2.CIR** | - | + | - | - |

**Supplementary Table 3. ALL markers used in flow cytometry gating strategy**
