# Supplementary material for: Cathepsin G Is Expressed by Acute Lymphoblastic Leukemia and Is a Potential Immunotherapeutic Target
Source: Front Immunol. 2018 Jan 25;8:1975. doi: 10.3389/fimmu.2017.01975 (PMC5790053; doi:10.3389/fimmu.2017.01975)
Supplement: Supplementary file 9 [file Table_4.docx]

| **Patient** | **A** | **B** | **C** | **DRB1** | **DQB1** | **DPB1** |
| --- | --- | --- | --- | --- | --- | --- |
| **UPN 5** | 02:05 | 08:01 | 07:01 | 03:01:01 | 02:01 | 03:01 |
|  | 01:01 | 39:01 | 07:01 | 01:01:01 | 05:04 | 03:01 |
| **UPN7** | 02:01 | 35:12:01 | 04:01 | 08:02:01 | 04:02 | 04:01 |
|  | 24:02 | 39:06:02 | 07:02 | 08:02:01 | 04:02 | 04:02 |

**Supplementary Table 4. HLA typing for patients used in the cytotoxicity assays.**
